# Design of Crystal Growth Dimensionality in Synthetic Wax: The Kinetics of Nonisothermal Crystallization Processes

**DOI:** 10.1021/acs.jpcb.3c05158

**Published:** 2023-10-03

**Authors:** Tomasz Rozwadowski, Łukasz Kolek

**Affiliations:** † Department of Chemical and Process Engineering, Faculty of Chemistry, Rzeszow University of Technology, 35-959 Rzeszow, Poland; ‡ Department of Materials Science, Faculty of Mechanical Engineering and Aeronautics, Rzeszow University of Technology, 35-959 Rzeszow, Poland

## Abstract

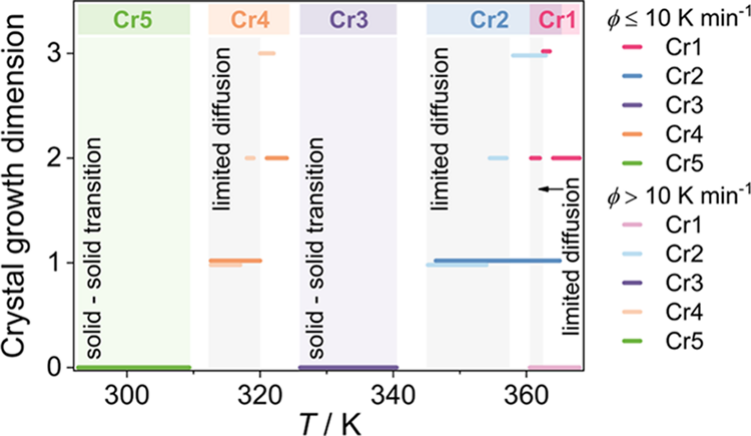

The demand for the development of multifunctional materials in emerging technologies has stimulated intensive research on the control of crystallization processes in numerous scientific and engineering fields. In this article, we examine the kinetics of nonisothermal melt crystallization in synthetic wax using differential scanning calorimetry (DSC) supported by polarized optical microscopy (POM) to describe crystallization modes in a multicomponent molecular system. We detected the macroscopic growth of three crystal phases and the formation of two crystal phases as a transformation from a disordered crystal mesophase into an ordered crystal. To characterize individual crystal phase formation, we examine the activation energy evaluated by isoconversional analysis and utilize the Ozawa and Mo methods to determine the kinetic details of the crystal growth from the isotropic phase. Our investigation reveals the possibility of the design of crystal growth dimensionality as three-dimensional spherulitic-like, two-dimensional rodlike, and one-dimensional needle-shaped crystal forms of shorter *n*-alkanes by controlling the solidification pathway of long-chain *n*-alkanes and the interplay of the thermodynamic and kinetic mechanisms of crystallization.

## 1 Introduction

In pursuit of novel materials design and efficient processing routes for emerging technologies, the control of crystallization in multicomponent systems is the key goal.^1−3^ In general, the development of new molecular materials with targeted physicochemical properties proceeds through a chemical way of synthesizing new molecules or by controlling the assembly of existing molecular components in the solid state. The dimensionality of crystallites appears as one of the essential factors determining the overall material properties and utility.^4−7^

Crystallization consists of two nonseparable processes, nucleation and then growth of the formed crystal nuclei to the macroscopic dimension, which are driven by thermodynamic and kinetic driving forces. Basically, the solidification path of the material is controlled by the overlap or separation of the rate curves of nucleation and growth. Upon cooling the melt, the former case promotes the aggregation of the molecules in the ordered structure of the crystal phase, whereas the latter leads to the formation of an amorphous solid.^8^ The crystalline solid as a mesophase may exhibit some degree of molecular disorder associated with conformational and orientational movements. Consequently, diverse molecular systems with various molecular shapes spanning from linear, globular, and rodlike to more complex were found capable of the formation of a disordered crystalline mesophase and its subsequent transformation to a more ordered crystal.^9−13^

Aggregation of molecules in multicomponent molecular materials may lead to the random location of different molecular constituents in equivalent crystallographic sites as an effect of the formation of mixed crystals (crystalline solid solutions). The molecular structure and interactions of individual components determine the ability to grow the crystalline solid phase(s). Chemically, similar molecules tend to form the mixed crystal phase, while the relevant difference in molecular design brings incompatibility in assembly and consequently crystallization of separate phases.^14−16^ The mixed crystal growth proceeds in miscellaneous multicomponent systems of diverse molecular weights and shapes, including pharmaceuticals and polymeric and wax materials, and the universal description of the process mechanisms is one of the prominent challenges of crystal engineering.^17−20^

Molecular assemblies of straight-chain (normal) *n*-alkanes (paraffins), derived from fossil fuels, form paraffin (petroleum) waxes.^21^ The crystallization of waxes is one of the emerging issues from a sustainable technology perspective in terms of both advantages and disadvantages. In the petroleum industry, the deposition of paraffin wax in crude tanks and pipelines affects flow efficiency at lower temperatures, increasing the demand for energy in transportation.^22^ On the other hand, wax crystallization is desired in the development of novel materials for energy storage in the energetics, electronics, and automotive industries.^23−26^ Moreover, waxes exhibit a strong hydrophobic nature and are commonly used as water repellents, protective layers, and surface modifiers.^27^ Controlling wax crystallization is crucial in industrial processes of casting, injection molding, 3D printing, sensor materials design, and pharmaceutical applications.^28−31^ For the reasons above, the exploration and modification of the crystallization behavior of waxes through various approaches become essential.^32−36^

In this paper, we investigate a commercial synthetic wax (BWM 101, Finish Kare).^37^ The material is widely used as a protective coating and includes long-chain *n*-alkanes of the desired higher melting point, compared to natural carnauba wax.^38^ Synthetic waxes essentially replicate characteristics of petroleum waxes while exhibiting higher chemical stability. Furthermore, the sustainable production process via Fischer–Tropsch synthesis provides independence from crude oil; thus, synthetic waxes become long-term alternatives.^39^ The chemical design of the material with *n*-alkane molecules of various lengths brings a capability for complex crystallization behavior and thus emerges as an object for the study of the expected interplay of proceeding crystallization processes. Moreover, the investigations of complex systems of *n*-alkane mixtures appear more desirable in industrial applications than pure single *n*-alkanes.^40^

The exploration of crystallization in waxes remains a challenging issue related to the general complexity of the system and the cross-linking of the crystals into the network structure, which creates severe limitations in structural investigations.^41^ Therefore, aiming to identify the mechanisms that control the crystal growth dimension at the molecular level, we employ the study of the kinetics of nonisothermal melt crystallization processes by differential scanning calorimetry supported by polarized microscopy displaying crystal morphology. The disclosed details of the formation of mixed crystal phases in the material improve the understanding of the solidification process of waxes, bridging a gap between experimental studies of pure or simple mixtures of *n*-alkanes and simulation approaches.

## 2 Experimental Section

### 2.1 Materials

The investigated material is a commercial synthetic hi-temp wax, BWM 101 (Finish Kare, USA). The material includes a mixture of low-molecular-weight aromatic hydrocarbons (1–10 wt %), solid paraffin (20–30 wt %), medium-molecular-weight alkanes (5–15 wt %), and high-molecular-weight alkanes (45–55 wt %).^37^

### 2.2 Methods

#### 2.2.1 Differential Scanning Calorimetry (DSC)

The thermal properties were examined by using a Netzsch DSC 214 Polyma (Germany) differential scanning calorimeter after a standard calibration procedure. The sample (7.2 mg) was sealed in an Al pan. Before each measurement run, the sample was heated to 393 K and kept for 3 min to ensure complete melting. Then, the sample was cooled down to 203 K with several constant rates in 1–30 K min^–1^ and subsequently heated up to 393 K with the same rate. The DSC data were analyzed after baseline correction and peak separation.

#### 2.2.2 Polarized Optical Microscopy (POM)

The microscopic observations were carried out at 203–393 K using a Leica DM2700 (Germany) polarized optical microscope with an Olympus (Japan) long-distance lens. The temperature was controlled by a Linkam THMS600 (U.K.) stage with a liquid nitrogen pump.

## 3 Results and Discussion

### 3.1 Crystallization Behavior

**Figure 1:**
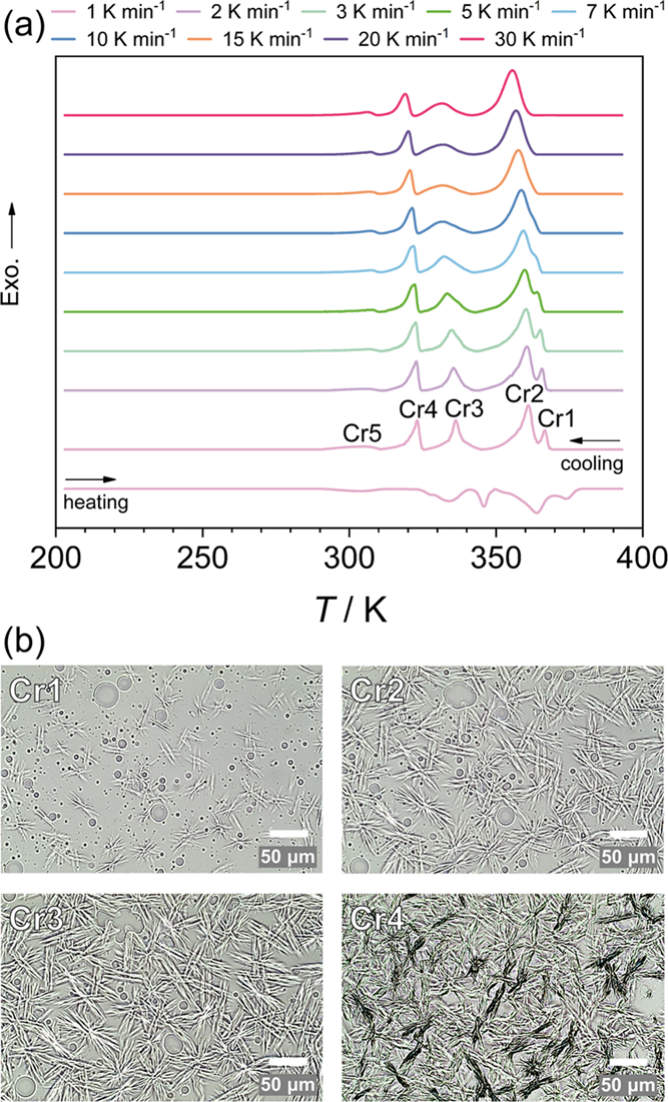
(a) DSC traces of synthetic wax (BWM 101, Finish Kare) in cooling and heating runs at various experimental rates. (b) POM micrographs of crystal formation processes; crystallization of Cr4 in the presence of Cr3 crystallites is shown after suppression of the crystallization of Cr1.

The crystallization behavior of synthetic wax (BWM 101, Finish Kare) was investigated through DSC and POM measurements at various temperature ramps. Figure 1 shows collected thermograms and microscopic textures. The material solidifies via multiple stages, a description of which we made while considering the general melting and phase transition behaviors of *n*-alkanes. According to the demonstrated trend, the melting point of *n*-alkanes increases with the molecular weight.^40^

To investigate phase transitions, we focus on exothermic anomalies of DSC, labeled in the order of their occurrence in cooling, as shown in Figure 1a. The first of the anomalies is related to the crystallization of heavy *n*-alkanes in the Cr1 phase and appears only at a low cooling rate, ϕ ≤ 10 K min^–1^. This behavior is related to the ease of supercooling of heavy *n*-alkanes in the multicomponent system. Therefore, the highest-weight molecules tend to form an amorphous state at ϕ > 10 K min^–1^, plausibly as an effect of the interaction of their long zigzag chains exhibiting a high degree of conformational and orientational disorder. Since the enthalpy of the transition (Table 1) is related to the mass amount of the formed crystalline phase, the crystal growth of Cr1 is rather moderate. The enthalpy of Cr1 crystallization is 10% of the total enthalpy associated with the growth of the crystalline phases (Cr1, Cr2, and Cr4) from the melt. It is noteworthy that long-chain *n*-alkanes with a high melting point do not exhibit a solid–solid transition,^21,42^ as also found for crystal Cr1.

**Table 1:** Peak Temperature and Enthalpy of Phase Transitions in DSC Heating Run

transition	*T*_trs_, K	Δ*H*, J g^–1^
melting of Cr1	375	2.0
melting of Cr2	364	12.2
Cr3–Cr2	346	3.0
melting of Cr4	335	6.1
Cr5–Cr4	304	1.6

The next crystallization process of Cr2 involves high-weight *n*-alkane molecules, with a bit shorter chains than that in the crystallization of Cr1. Deducing from the transition enthalpy, the crystalline phase of Cr2 is predominant, with 60% of the total mass of the crystalline phases in the solidified material. Subsequent anomaly applies to the crystal–crystal transition, i.e., from the crystalline rotator phase Cr2 (orientationally disordered crystal) to the ordered crystal Cr3. Typically, this transition is expected for the crystalline mesophase of *n*-alkanes at this temperature range and is associated with the suppression of thermally activated rotation along the long axis of the molecule.^21^

Succeeding anomalies reveal the same nature as higher-temperature ones, i.e., they are related to the formation of the crystalline mesophase, Cr4, and its transformation to the ordered crystal, Cr5. These transitions apply to shorter *n*-alkane molecules (compared to those involved in the crystallization of Cr1 and Cr2) and fall well within the temperature region of the typical paraffin wax crystallization.^21^ As expected, the enthalpy ratio of the solid–solid transitions Cr2–Cr3 and Cr4–Cr5, Δ*H*_Cr3_/Δ*H*_Cr5_, is the same as the enthalpy ratio associated with the growth of the Cr2 and Cr4 phases, Δ*H*_Cr2_/Δ*H*_Cr4_.

To support the phase behavior investigation and identify the crystal morphology, we applied POM observations. Figure 1b shows the microscopic textures captured during cooling from the melt. The growth of crystal Cr1 is relatively small, and the large (dark) regions of the disordered state, which does not change the light polarization plane, are recognized. Further cooling triggers the crystallization of Cr2, which yields a relatively high amount of the crystal phase. During the crystal formation of Cr3, no crystallite growth is observed, which confirms that this is the solid–solid transition and not macroscopic growth. The analogous observation applies to the crystal formation of Cr4 and Cr5; the former is associated with the growth of the crystalline phase, while the latter does not, as displayed in the texture pattern. Relatively large areas of the amorphous regions found in microscopic images and a low transition enthalpy evaluated from DSC imply that the crystallization exhibits limitations related to the molecular interaction and low compatibility between alkane molecules as an effect of the wide distribution in a complex system.^43^ Consequently, overall crystallinity is moderate compared to a simple paraffin wax exhibiting two crystalline phases.^44,45^ At this point, it should be stressed that the aggregation and dispersion of the amorphous state produce local fluctuations in density, significantly involved in the crystallization process.^43^

The POM images display that crystallites of the Cr1 phase show mostly rodlike morphology and sporadically spherulitic-like forms at slow cooling. After crystallization of the longest-chain molecules, the shorter molecules form networks with rod- and needle-shaped crystals of the Cr2 and Cr4 phases. In contrast, suppression of crystallization of Cr1 in fast cooling also promotes the formation of larger crystallites of Cr2 and Cr4 with three-dimensional forms that resemble spherulitic structures.

**Figure 2:**
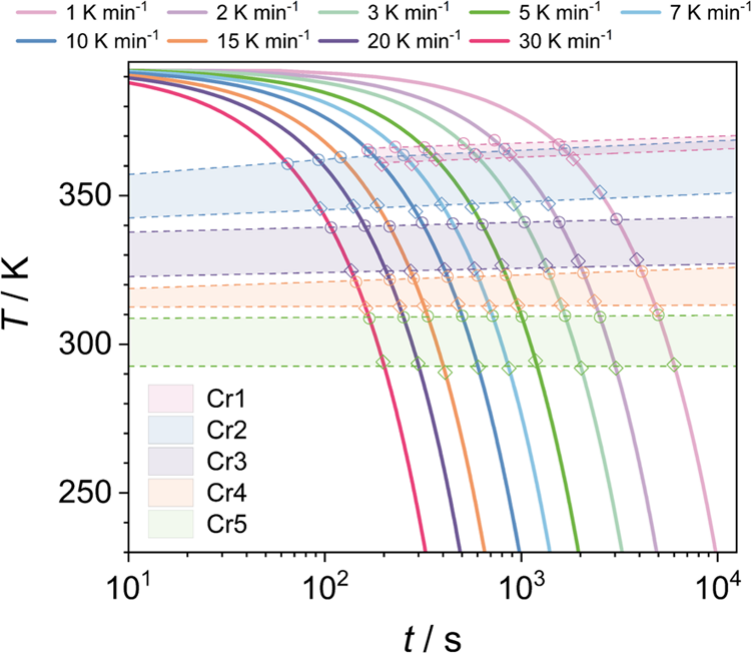
Continuous cooling transition (CCT) diagram. The onset of the phase transition is denoted by circles, and the endset by diamonds. The dashed lines show the process borders.

Further insights related to the kinetic nature of the crystallization can be drawn from the continuous cooling transition (CCT) diagram shown in Figure 2. Namely, the CCT diagram locates the phase transition processes on a time–temperature scale. The estimated borders for the region where phase transitions proceed reveal two trends. Specifically, processes related to the growth of the crystalline phase in the material show a strong dependence on the cooling rate, in contrast to the crystal–crystal transformations.

### 3.2 Nonisothermal Crystallization Kinetics

**Figure 3:**
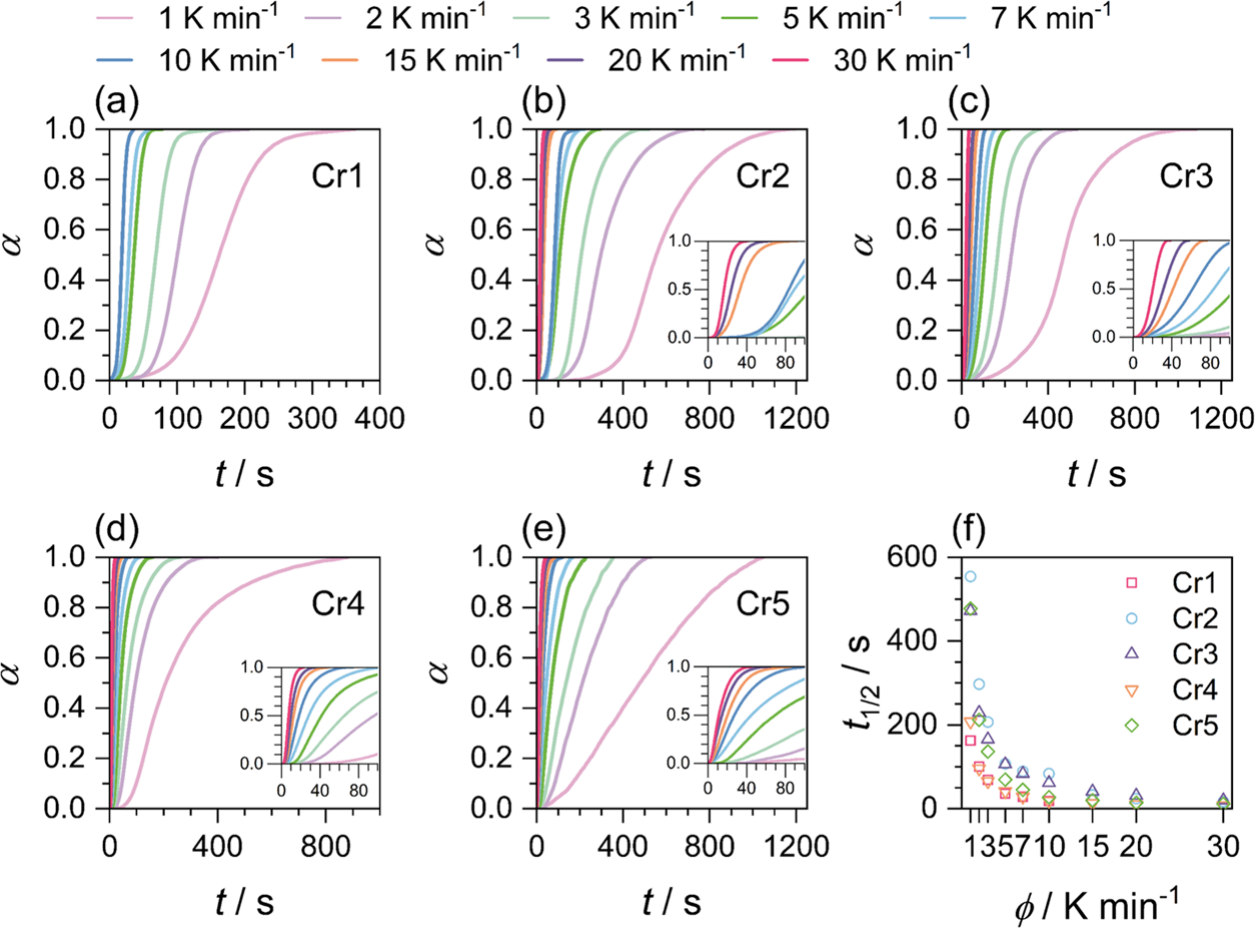
(a–e) Time evolution of the relative crystallinity degree α for individual crystal formation processes; insets show the beginning of the process. (f) Half-time of the process *t*_1/2_ as a function of the cooling rate ϕ.

The description of the underlying molecular-level mechanisms that control the crystallization events in the material can be drawn through an investigation of the kinetics of processes by combining complementary approaches. First, to characterize the phase transitions, including crystal growth and solid–solid transitions, proceeding at various cooling ramps, we examine the relative degree of conversion based on the detected anomalies from Figure 1a. For this purpose, we analyze the phase transitions as separate processes and determine the relative crystallinity degree α(*T*) as the relative extent of conversion from the initial to the final stage of the individual phase transformation process under nonisothermal cooling conditions. The relative crystallinity (conversion) degree α(*T*) is calculated as the fraction of the mass of the transformed sample from the enthalpy change in the process
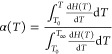
1where d*H*/d*T* is the heat flow rate, *T*_0_ denotes the start point of the examined process, and *T*_∞_ is the endpoint. Since the applied cooling rate ϕ is constant, the determination of the time evolution of the degree of crystallinity is based on the following relation
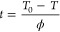
2Figure 3a–e shows the degree of conversion for the processes of each crystal phase formation, including macroscopic growth (Cr1, Cr2, and Cr4) and the solid–solid transition (Cr3 and Cr5) as a function of time, α(*t*), at various cooling rates ϕ. In Figure 3f, the half-time of crystal formation as a function of the cooling rate is presented. It is clearly noticeable that the processes follow the general trend of acceleration with increasing ϕ. The crystal growth of Cr2, which contributes most to the total crystallinity, proceeds for longer than other processes.

To discuss the variation in mechanisms driving the individual steps of crystal formation, we start with the determination of the effective activation energy. The activation energy of crystal formation is assumed as the energy required for the formation of crystal nuclei exceeding the critical size, which leads to the completion of the nucleation and initiates the growth, or the energy necessary to overcome the barrier associated with the suppression of molecular rotation in the transformation process of a disordered crystal to an ordered crystal.^46^ The positive or negative energy determines the distinct temperature regions, where the process is mainly controlled by molecular diffusion or thermodynamic driving force, respectively. Typically, crystallization at low supercooling is expected to proceed as a thermodynamically controlled process.^8,47^

The activation energy of crystallization is contributed by the molecular structure and interactions, as well as the difference in the positional and conformational order of molecules between the initial phase and the forming crystal, and depends on thermodynamic and spatial conditions. In particular, molecular flexibility brings the additional activation barrier for crystal formation.^48,49^ Therefore, high-weight flexible molecules exhibiting backbone conformations are prospective for a lower capability for melt crystallization than small ones.^50^ Additionally, in the wax material, a broad distribution of *n*-alkanes also favors an increase in the activation barrier for crystallization and thus the tendency to form an amorphous state.

To calculate the effective activation energy through the isoconversional method, we utilize the Friedman equation as follows^47,51^
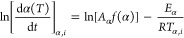
3where index *i* identifies an individual cooling rate ϕ, and *T*_α,i_ is the temperature to reach a given crystallinity α, *A*_α_ is a constant, *f*(α) is a reaction model, *R* is a gas constant, and *E*_α_ is the effective activation energy of the process. The activation energy *E*_α_ is determined as a slope of the plot of the logarithm of dα(*T*)/d*t* at the respective relative crystallinity degree α and cooling rate ϕ versus reciprocal temperature (1/*T*_α,i_) corresponding to specified α and ϕ. Furthermore, to assess the dependence of crystallization mechanisms on temperature, we also consider energy *E*_α_ as a function of average temperature *T*_avg_.

**Figure 4:**
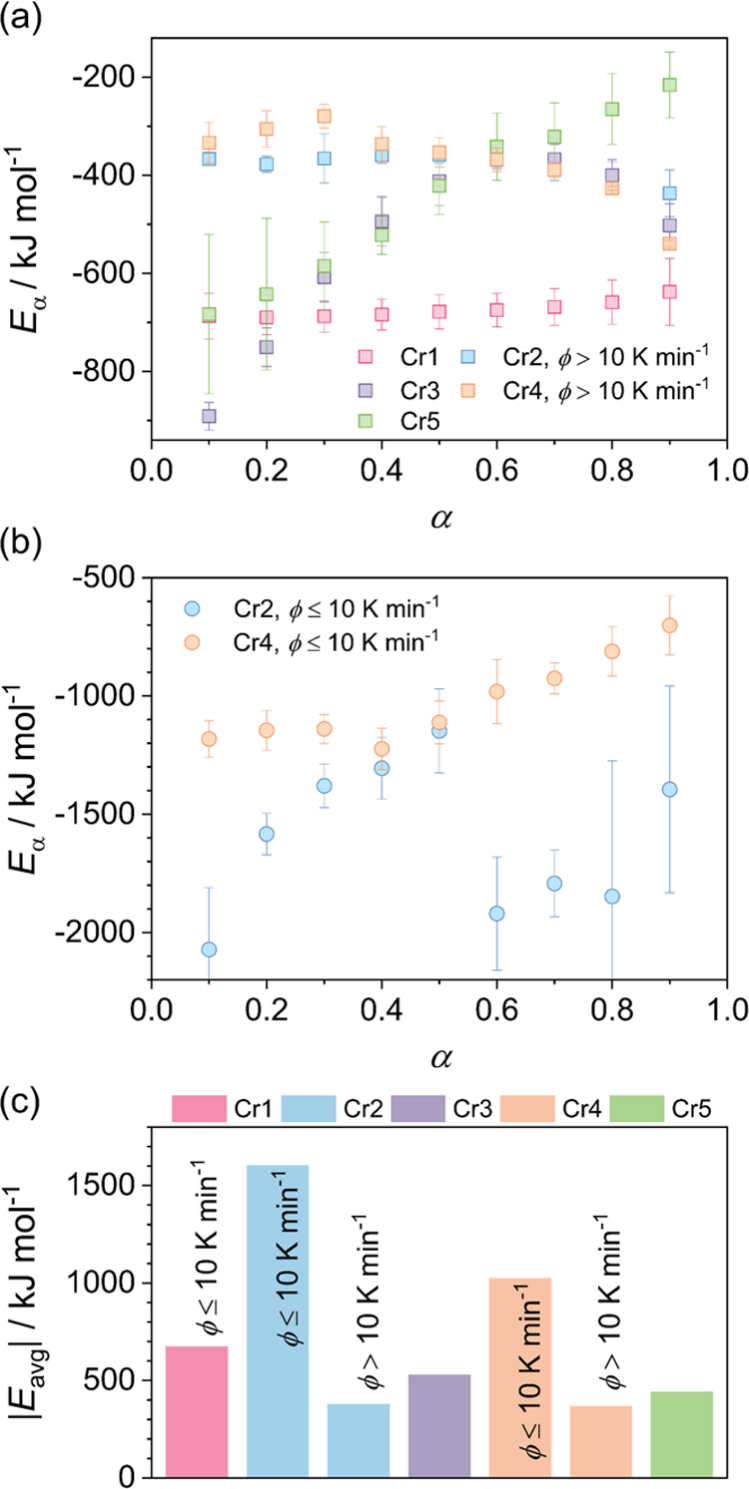
(a, b) Effective activation energy *E*_α_ as a function of the relative crystallinity degree α. (c) The magnitude of the average activation energy |*E*_avg_| for individual processes. The influence of the cooling rate on the crystallization of Cr2 and Cr4 is shown.

**Figure 5:**
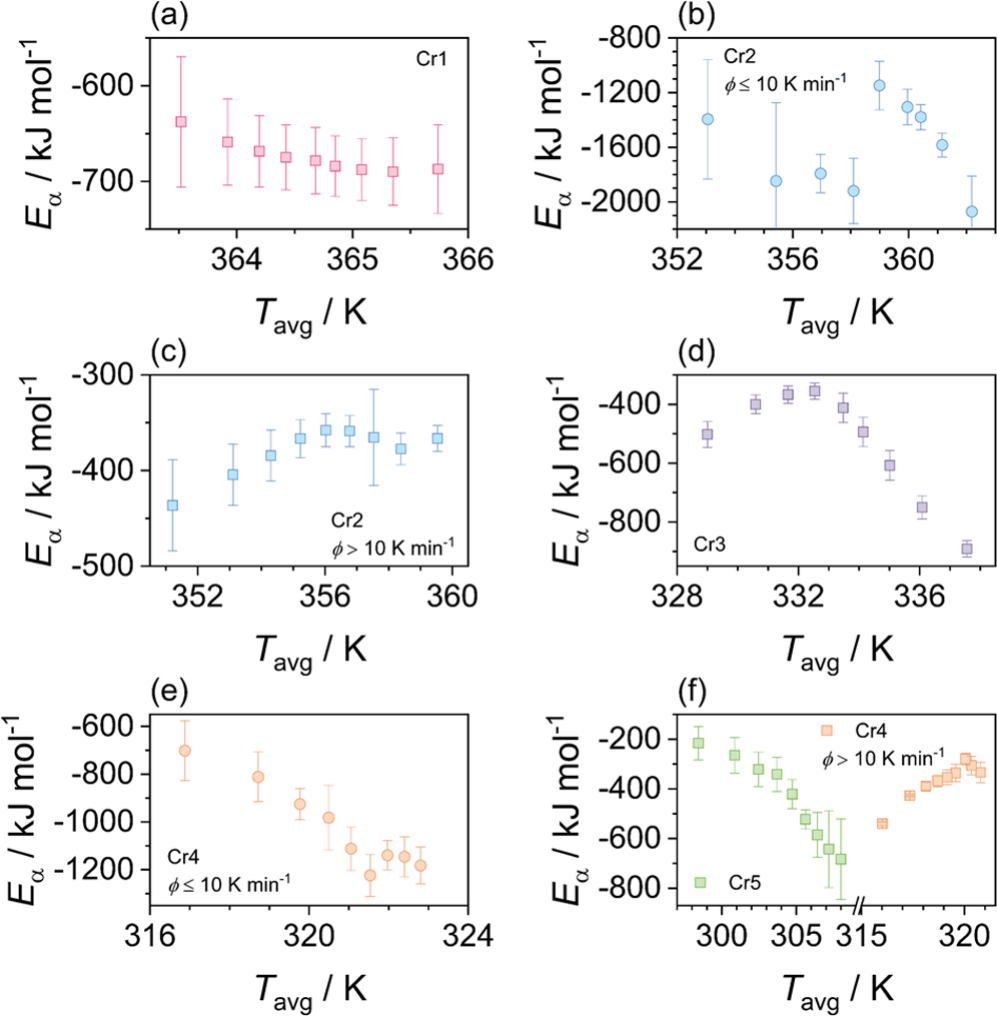
(a–f) Effective activation energy *E*_α_ as a function of the average temperature *T*_avg_ corresponding to the relative crystallinity degree determined for individual crystal formation processes.

Figures 4 and 5 show the dependence of the effective activation energy on the relative degree of crystallinity and temperature, respectively. In general, the activation energy exhibits explicit variation in both the change direction and the value range (averaged in Figure 4c). The determined values of *E*_α_ are negative for each process, implying that the crystallization events in the material are mainly driven by the thermodynamic driving force. However, the registered trend changes point to the appearing role of the molecular mobility factor and thus to the interplay of both crystallization drives at higher conversion degrees at lower temperatures.

Crystallization of the Cr1 phase exhibits a tendency to suppression at high cooling rates (ϕ > 10 K min^–1^), resulting in formation of the amorphous instead of the crystalline state. Therefore, the absolute values of the activation energy of this process are relatively high. For crystallization processes of Cr2 and Cr4, the activation energy shows the relationship with the cooling rate ranges revealed in separate dependencies for cooling rates lower or higher than 10 K min^–1^ (Figure S1), related to the solidification behavior of the longest-chain *n*-alkanes. When the crystallization processes of Cr2 and Cr4 proceed at low cooling rates and growth is affected by the crystal Cr1 already formed from the longest molecules, *E*_α_ shows a noticeably higher magnitude. Once the suppression of crystallization of Cr1 happens, this removes an additional energy barrier related to molecular steric constraints for the crystal growth of Cr2 and Cr4, and thus, a significant decrease in |*E*_α_| appears. At half of the conversion, the magnitude of the activation energy for the crystallization of Cr2 and Cr4 is similar and exhibits a decrease by the same factor at cooling rates higher than 10 K min^–1^, compared to lower cooling rates. This points out that the Cr1 crystallization affects the spatial conditions for the Cr2 and Cr4 growth equally at this stage.

The activation energy of the Cr2 and Cr4 crystallization processes is comparable at high cooling rates, and at slow cooling, a distinct difference emerges at a conversion degree higher than 0.5, as shown in Figure 4. Namely, this finding is related to the change in the process mechanism. As to the principle, a share of molecular diffusion in the overall crystallization mechanism intensifies at lower temperatures, i.e., below 358 K for the crystallization of Cr2 and 320 K for the crystallization of Cr4 in the present case (see Figure 5). Interestingly, the breakouts of the trends proceed in different directions related to the crystal phase and the cooling rate. The values of *E*_α_ remain negative, which implies the limitation in molecular diffusion. These effects can be plausibly connected with spatial constraints and crystal morphology, as discussed further below. A similar effect of the negative effective activation energy of the melt crystallization process in the temperature region controlled by the kinetic driving force has also been found for the low-weight mesogenic substance.^52^ In the investigated material, the magnitudes of the activation barrier are reasonably higher than for neat systems of small molecules, and at higher cooling rates, when the Cr1 crystallization is suppressed, they are within a comparable range to typical paraffin wax.^44^ The solid–solid transition shows a slightly higher activation barrier for longer-chain molecules that form the Cr3 phase compared to the Cr5 phase.

Based on the crystallization mechanism pattern drawn up through the effective activation energy analysis, we proceed to a further detailed description of the interplay of crystallization driving forces and the effect on crystal morphology by employing kinetic models. One of the most commonly utilized methods to analyze the kinetics of crystallization under isothermal conditions is the Avrami model^53^
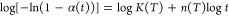
4where *n* is the Avrami exponent linked to the growth dimensionality and mode of crystallization, and log *K* is the crystallization rate parameter. Crystal nucleation and growth rates strongly depend on temperature, and thus, the determination of crystallization parameters under nonisothermal conditions requires adjustment. Ozawa used the Evans derivations^54^ to the Avrami model (eq 4) to arrive at the following equation that enables the examination of the process at constant experimental rates ϕ^55^
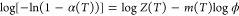
5where *m* is the so-called Ozawa exponent, equivalent to the Avrami exponent *n*, and log *Z* is the crystallization rate parameter. The application of the Ozawa model by plotting log[−ln(1 – α(*T*))] against log ϕ at a given temperature enables us to determine index *m* and parameter log *Z* from the slope and intercept, respectively.

**Figure 6:**
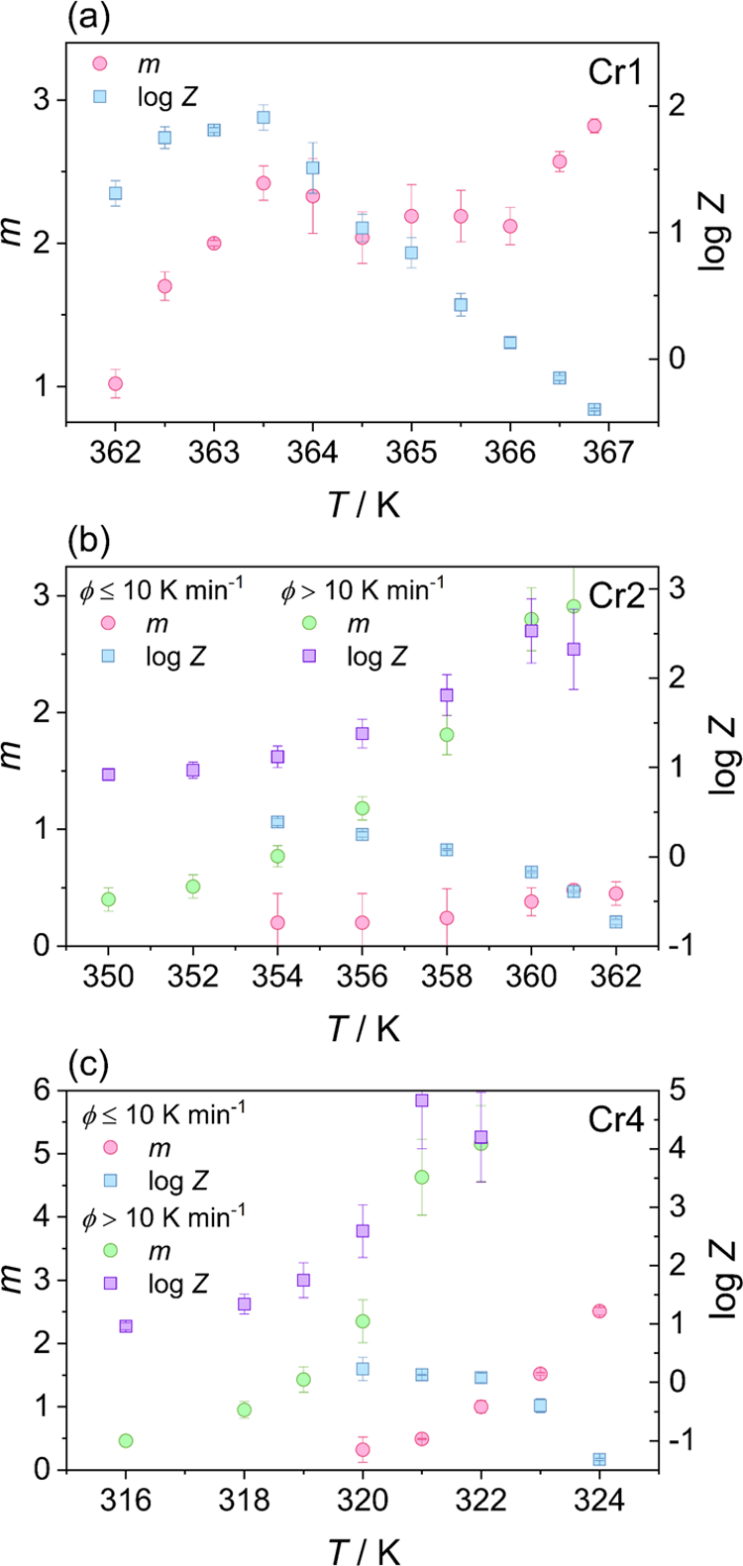
Ozawa exponent *m* and crystallization rate parameter log *Z* as a function of temperature for the crystal growth process of (a) Cr1, (b) Cr2, and (c) Cr4.

Figure 6 shows the parameters determined by the Ozawa method applied for the crystallization processes of Cr1, Cr2, and Cr4. The analysis is not feasible for the crystal–crystal transformation due to the nature of the transition related to the suppression of molecular movements and not macroscopic crystal growth. Plots of log[−ln(1 – α(*T*))] versus log ϕ exhibit single linear dependence for the Cr1 crystallization process, while for Cr2 and Cr4, two dependencies appear in the cooling rate ranges separated at 10 K min^–1^ (Figure S2).

We consider index *m* (eq 5) under the assumption of mutual equivalency of the exponents from the Avrami and Ozawa methods. Based on this, the relation that involves the contribution of the factors related to the nucleation process and the dimension of the crystal growth stands
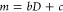
6where *b* is the growth mode index (*b* = 0.5 for diffusion growth, *b* = 1 for thermodynamic growth), *D* is the growth dimensionality, and *c* is the nucleation index (*c* = 0 for the zero nucleation rate, 0 < *c* < 1 for the decreasing nucleation rate, *c* = 1 for the constant nucleation rate, and *c* > 1 for the increasing nucleation rate). Under the aforementioned assumptions, we advance to the description of crystal growth by combining the evaluation of index *m* and crystallization rate parameter log *Z* with the activation energy dependencies.

The examination of the crystallization of Cr1 by the Ozawa method (eq 5) reveals that parameter *m* roughly scatters around 2.5, and crystallization rate parameter log *Z* exhibits a constant increase at cooling, down to 363.5 K. This divergence between the crystallization rate and molecular mobility, which is hindered by increasing viscosity in lowering the temperature, implies that the process is thermodynamically driven in this temperature range. Thus, according to eq 6, crystal Cr1 growth at temperatures higher than 363.5 K proceeds in two dimensions with a decreasing nucleation rate. Below the breakpoint temperature of 363.5 K, parameter log *Z* starts to decrease instantaneously, exposing a coupling between the crystallization rate and molecular mobility. This reveals the change in the crystallization mechanism, i.e., the transition to the temperature region controlled by the kinetic mechanism. However, considering the negative activation energy, the thermodynamic mechanism shows prevalence, pointing to the significant barrier for molecular diffusion drive. The values of index *m* and POM observations indicate a growth mode transition from initially two-dimensional rodlike crystals to large three-dimensional spherulite-like aggregates just below 363.5 K and then back to the rodlike structures at 362 K. In continuous cooling, rod-shaped crystals grow along radial directions and then tend to align densely into a spherulitic-like structure. Subsequently, the process evolves to the two-dimensional growth of rod-shaped crystallites due to the limitation of molecular mobility and can also be plausibly connected with the initiation of the growth of the crystal phase Cr2.

The growth of the Cr1 phase (at ϕ ≤ 10 K min^–1^) or its suppression (at ϕ > 10 K min^–1^) determines the conditions for the crystallization process of Cr2. As deduced from the temperature dependence of index *m*, a steric hindrance arising from the presence of crystallites of Cr1 significantly affects the growth dimension of the Cr2 phase. Namely, Cr2 growth is then one-dimensional in needle-shaped forms, which produces networks between crystallites. The suppression of crystallization of Cr1 favors the three-dimensional spherulitic-like growth of Cr2 accompanied by a significant decrease in the activation barrier (Figure 4c). However, the limitation in the crystal growth dimension of Cr2 gradually progresses at cooling; the growth evolves from three, through two (below 358 K), down to one dimension at temperatures lower than 354 K. A low strength of the molecular diffusion mechanism appears to be the essential factor determining the change in crystal morphology at temperatures below the reversal of the trends of log *Z*(*T*) (Figure 6b) and *E*_α_(*T*) (Figure 5b,c). A high barrier for molecular mobility at temperatures lower than 358 K is reflected in the (negative) values of activation energy and apparent prevalence of the thermodynamic mechanism, resulting in the limitation of growth dimensionality.

The crystallization of Cr4 proceeds in modes similar to those of Cr2 and displays two pathways related to the crystallization behavior of the heaviest *n*-alkanes in phase Cr1, determined by cooling rates. The process exhibits the change of the decisive factor in the crystallization mechanism. Crystal Cr4 grows in two modes, separated at around 321 K, as shown in Figure 6c. The change in activation energy also supports this finding (Figure 5e,f). At temperatures higher than 321 K, growth remains mainly driven by the thermodynamic driving force, whereas at lower temperatures, the limited contribution of the kinetic drive becomes crucial. During slow cooling, ϕ ≤ 10 K min^–1^, despite high space constraints from the existing crystallites of the Cr1 and Cr3 phases, the shorter molecules aggregate initially in two dimensions as rodlike crystals of the Cr4 phase, as deduced from index *m*. At lower temperatures, the growth of Cr4 is limited to one dimension due to the decaying kinetic driving force in the viscous liquid. The complete suppression of crystal Cr1 formation brings the conditions for the three-dimensional growth of crystal Cr4, which is expectantly gradually reduced due to low molecular mobility in lower-temperature regions to two dimensions below 320 K and finally to one dimension at around 316 K.

The revealed differences in the evolution of the Cr2 and Cr4 crystal morphologies at the diffusion-limited temperature regions in slow cooling can be rationalized based on the activation energy dependencies displayed in Figure 5. The change in the Cr2 crystallization mechanism to the limited diffusion drive leads to one-dimensional growth at low temperatures and is manifested in a jump in the activation barrier, |*E*_α_|. On the contrary, during Cr4 crystallization, |*E*_α_| shows a progressive decrease. This reasonably indicates the ability for higher mobility of smaller molecules to form the Cr4 phase, which aggregate mostly in two dimensions between the crystallites of the Cr3 phase.

**Figure 7:**
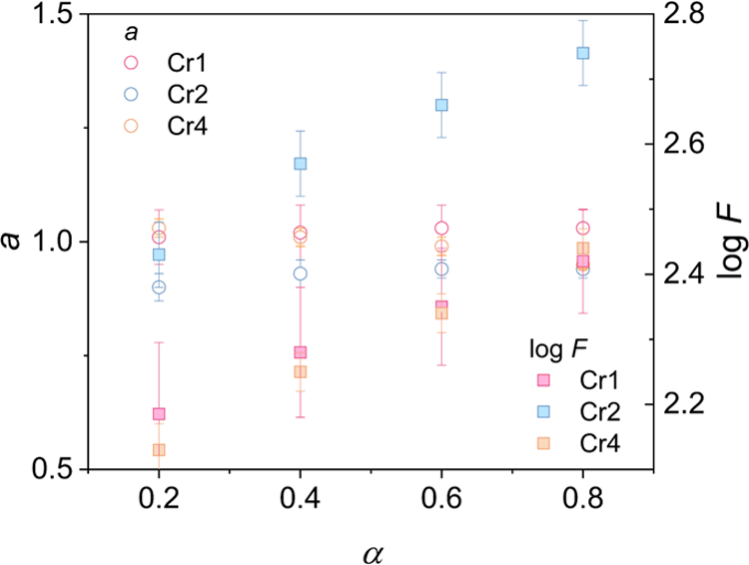
Crystallization parameters *a* and log *F* from the Mo model as a function of the relative crystallinity degree α.

Additionally, to further illustrate the relation between the cooling rate and time of macroscopic crystal growth on the background of revealed crystallization mechanism patterns, we employ the model developed by Liu and Mo.^56^ This approach lies in the combination of the right-hand side of the Avrami (eq 4) and Ozawa (eq 5) equations, resulting in the following form
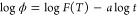
7where the parameter *F*(*T*) = [*Z*(*T*)/*K*(*T*)]^1/*m*^ represents the cooling rate required to achieve certain crystallinity within the time unit and is inversely proportional to the crystallization rate, and *a* is the ratio of the Avrami exponent *n* to the Ozawa exponent *m*. The plots of log ϕ versus log *t* at a given crystallinity show a linear relationship that yields parameters *a* and log *F* from the slope and intercept, respectively (Figure S3). The determined parameters are listed in Figure 7. Parameter *a* reasonably scatters around 1, and log *F* increases gradually with proceeding crystallization, exhibiting different value ranges depending on the crystal growth process. Parameter log *F* indicates that the crystallization of Cr2 is a notably slower process than that of Cr1 and Cr4, which proceed at a similar rate. This is in agreement with the crystallization half-time analysis. Furthermore, the investigated processes expose a common feature of slowing down with increasing crystallinity and deeper supercooling, accompanied by a limitation in the crystal growth dimension related to hindered molecular diffusion at lower temperatures.

Recently, it has been demonstrated that the mechanical properties of wax are correlated with crystal morphology. Spherulitic structures induce low mechanical strength, whereas small crystals significantly improve it.^45^ In view of this report, the revealed ability to control the solidification path of the heaviest molecules and the strength of the molecular diffusion drive, which determine the crystal growth dimension, allows tuning of the hardness of the material. In the investigated material, after the crystallization of long-chain molecules in the Cr1 phase, the shorter molecules form crystallite bridges in the available space, thereby implying an increase in mechanical strength. On the contrary, the suppression of Cr1 crystallization through fast cooling, followed by a slow cooling protocol in a thermodynamically controlled process, yields the formation of spherulitic-like structures with weak mechanical connections between crystallites, which suggests increasing plasticity.

## 4 Conclusions

We investigate the crystallization behavior and mechanisms controlling the dimensionality of crystal growth in synthetic wax under continuous cooling conditions. The aggregation of *n*-alkane molecules in the investigated material proceeds as a macroscopic growth of three crystalline phases, of which two lower-temperature phases of shorter molecules exhibit a further solid–solid transition. The revealed facility of switching between the crystallization of the longest molecules in the system and its suppression brings a viable route to control the spatial constraints that contribute to the hindrance of the aggregation of shorter molecules. Moreover, the primary mechanism of crystal growth processes exhibits a transition between temperature regions controlled by thermodynamics and kinetics, and the mechanistic insights revealed that the latter drive plays a key role in tuning the crystal morphology. Namely, the decaying molecular mobility at low temperatures is accompanied by the apparent prevalence of the thermodynamic mechanism, reflected in the negative activation energy, which results in a limitation of the growth dimension.

Overall, we find that the crystal growth dimensionality is affected by two essential factors: the solidification path of the longest molecules and the interplay of the crystallization driving forces. The investigation shows that both factors can be controlled through the cooling protocol, which yields the capability to design the crystal morphology in three-, two-, and one-dimensional forms. In conclusion, the combined kinetic approaches provide a glimpse of crystallization in a multicomponent *n*-alkane system and reveal a way to tune the molecular-level mechanism that controls the macroscopic features of the wax material.

## Supporting Information Available

Kinetic analysis based on the Friedman, Ozawa, and Mo methods (Figures S1–S3) (PDF)
