# Supplementary material for: Design of Crystal Growth Dimensionality in Synthetic Wax: The Kinetics of Nonisothermal Crystallization Processes
Source: J Phys Chem B. 2023 Oct 3;127(40):8697–705. doi: 10.1021/acs.jpcb.3c05158 (PMC10578349; doi:10.1021/acs.jpcb.3c05158)
Supplement: Supplementary file 1 — jp3c05158_si_001.pdf [file jp3c05158_si_001.pdf]

Supporting Information

Design of Crystal Growth Dimensionality in Synthetic Wax: The Kinetics of Nonisothermal Crystallization Processes

Tomasz Rozwadowski*[1] and Łukasz Kolek[2]

[1] Department of Chemical and Process Engineering, Faculty of Chemistry,

Rzeszow University of Technology, 35-959 Rzeszow, Poland

[2] Department of Materials Science, Faculty of Mechanical Engineering and Aeronautics,

Rzeszow University of Technology, 35-959 Rzeszow, Poland

*Email: tomasz.rozwadowski@prz.edu.pl

Contents

Figure S1. Isoconversional method applied to the crystallization of (a) Cr2 at crystallinity degree $\alpha = 0.3$ and (b) Cr4 at $\alpha = 0.3$.

Figure S2. Ozawa plot for the crystallization of (a) Cr1 at $T = 365.5$ K, (b) Cr2 at $T = 360$ K, and (c) Cr4 at $T = 320$ K.

Figure S3. Mo method applied to the crystallization of Cr1 at various degrees of crystallinity α.

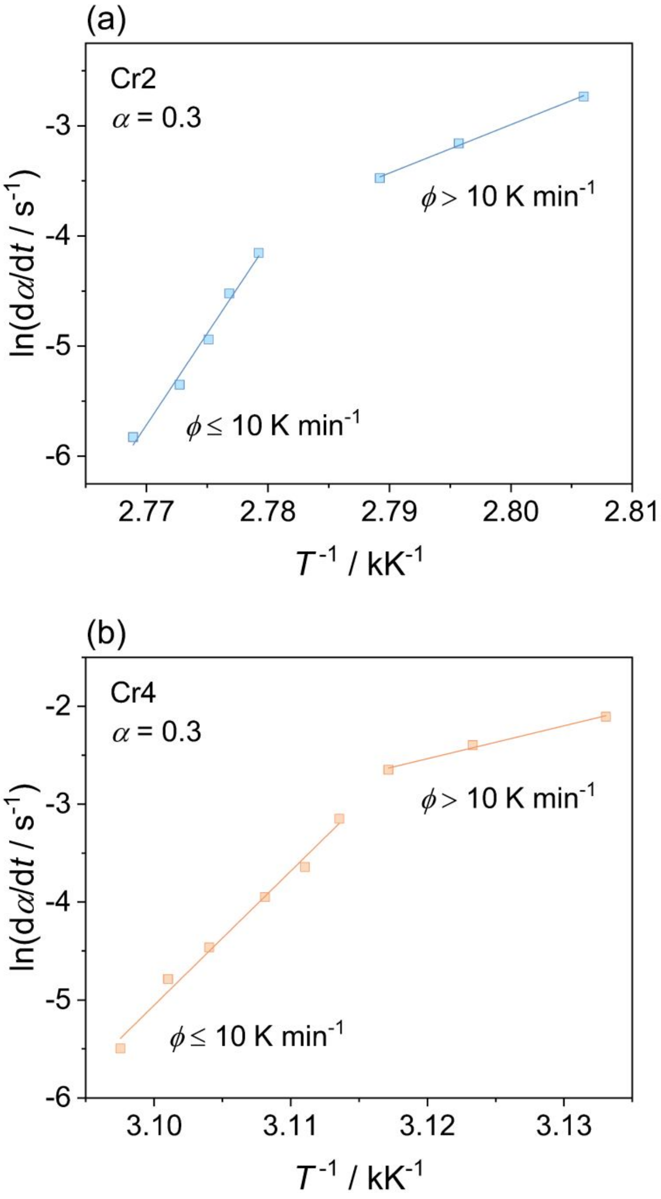

Figure S1. Isoconversional method applied to the crystallization of (a) Cr2 at crystallinity degree $\alpha = 0.3$ and (b) Cr4 at $\alpha = 0.3$.

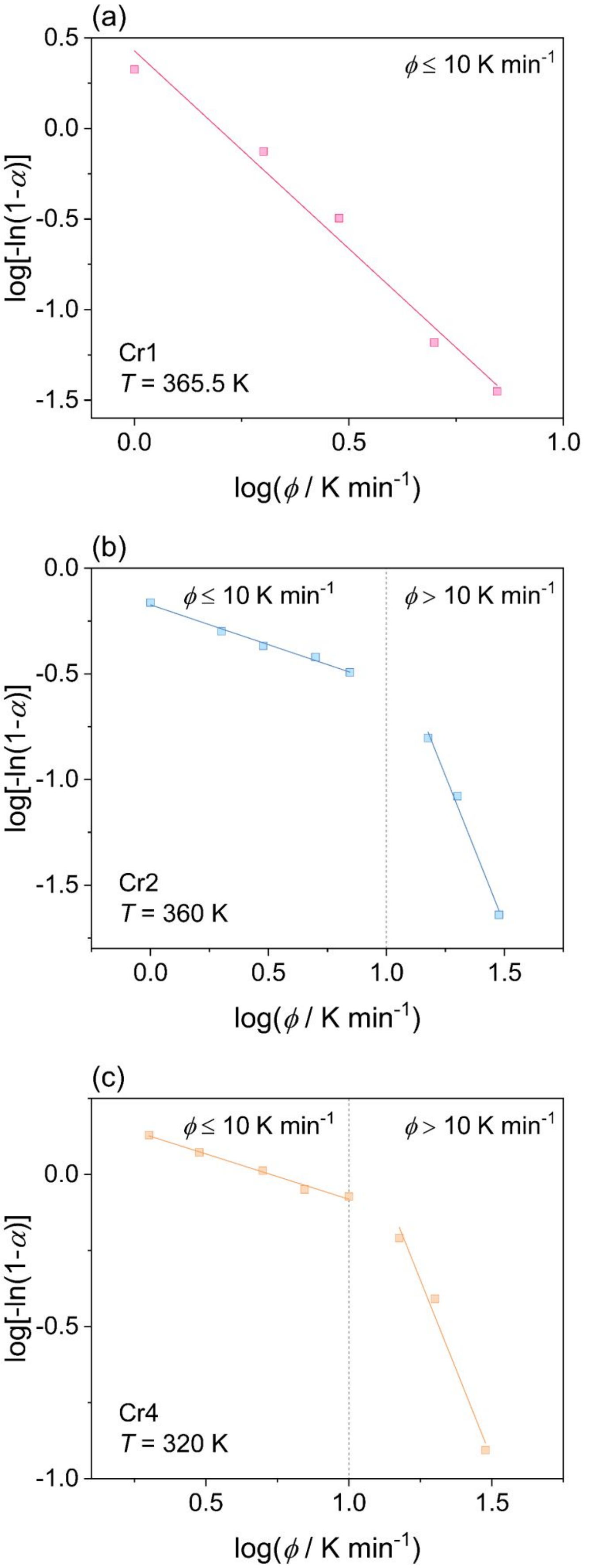

Figure S2. Ozawa plot for the crystallization of (a) Cr1 at T = 365.5 K, (b) Cr2 at T = 360 K, and (c) Cr4 at T = 320 K.

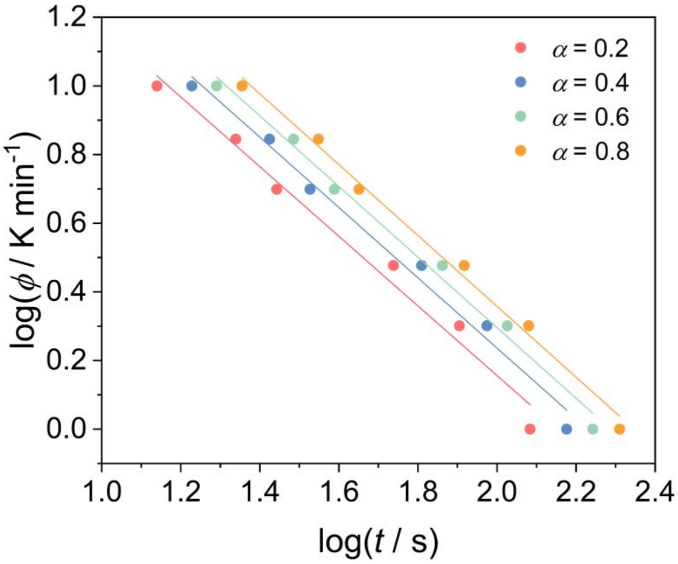

Figure S3. Mo method applied to the crystallization of Cr1 at various degrees of crystallinity α.
